# Supplementary material for: Gut microbiota-mediated conversion of mangiferin to norathyriol alters short chain fatty acid and urate metabolism
Source: Gut Microbes. 2025 May 22;17(1):2508422. doi: 10.1080/19490976.2025.2508422 (PMC12101588; doi:10.1080/19490976.2025.2508422)
Supplement: Supplemental Material [file KGMI_A_2508422_SM7434.zip › Bunt et al Suppl figures.docx]

**Gut microbiota-mediated conversion of mangiferin to norathyriol alters short chain fatty acid and urate metabolism**

**Authors:**

Daan Bunt^1,3*^, Markus Schwalbe^1,2*^, Fittree Hayeeawaema^1^, Sahar El Aidy^1,2#^

**Affiliations:**

^1^Host-Microbe Interaction, Groningen Biomolecular Sciences and Biotechnology Institute (GBB), University of Groningen, 9747 AG Groningen, The Netherlands.

^2^Microbiome Engineering, Microbiology Department, Swammerdam Institute for Life Sciences (SILS), University of Amsterdam

^3^Stratingh Institute for Chemistry, University of Groningen, Nijenborgh 7, 9747 AG Groningen, The Netherlands.

**# Corresponding author:**

Swammerdam Institute for Life Sciences, University of Amsterdam, Science Park 904, 1098 XH Amsterdam, Netherlands. e-mail address: [s.elaidy@uva.nl](mailto:s.elaidy@uva.nl)

***** Shared first author

**Supplementary Figure 1**. (**A**) HPLC-UV chromatograms anaerobic fermentation of MAN (measuring at 256 nm). (**B-C**) HPLC-UV chromatograms aerobic fermentation of MAN, using either EBB or carbohydrate-deprived EBB (measuring at 256 nm). (**D**) LCMS chromatograms and mass spectra of anaerobic fermentation of MAN, in case of partial conversion. The UV-absorption peak of MAN is at 2.30 min with [M+H]+ of 422.92, and of NOR at 5.57 min with [M+H]+ of 261.04.


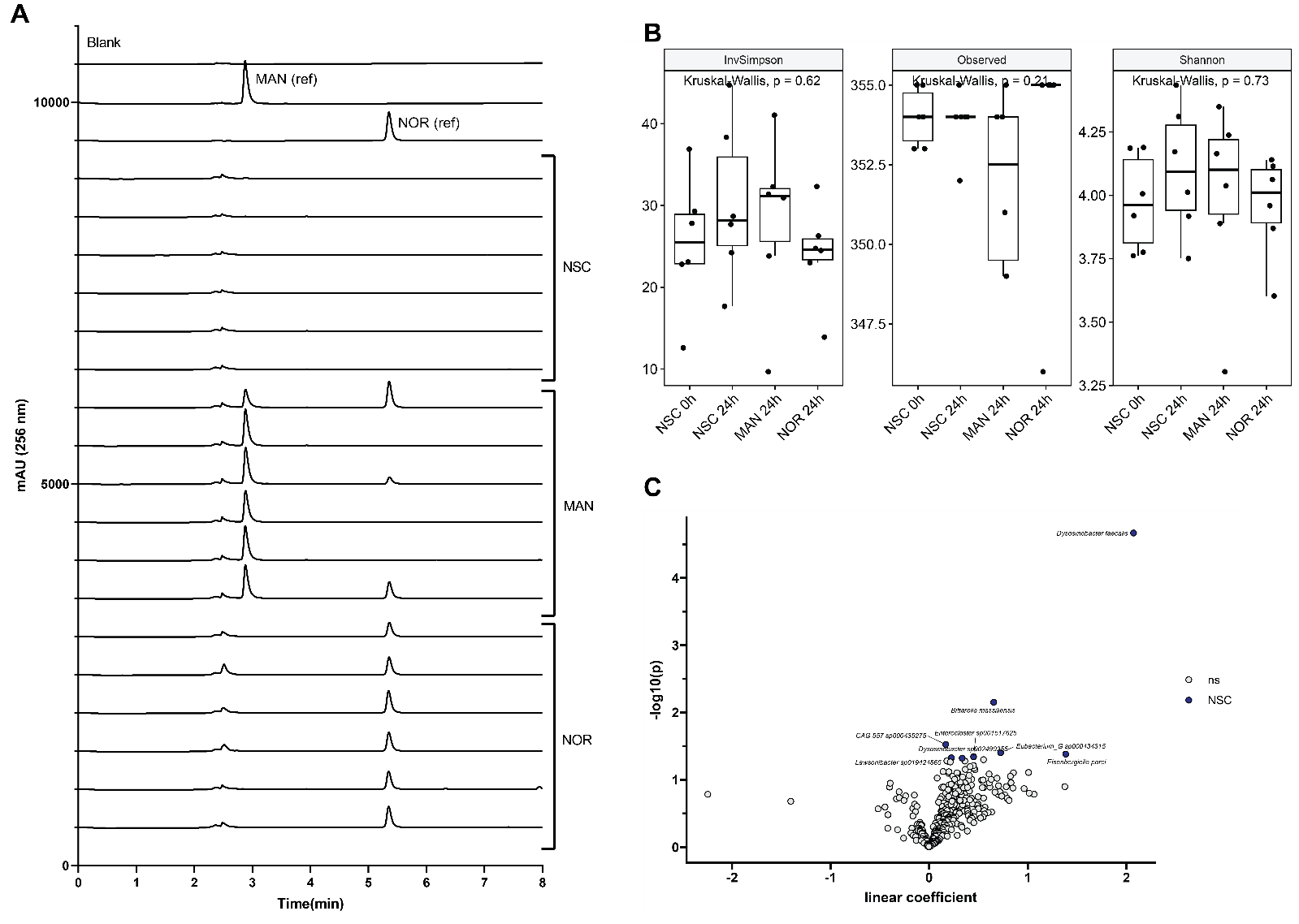


**Supplementary Figure 2**. (**A**) HPLC-UV chromatograms SIFR® fermentation of MAN, NOR, or no substrate (NSC) (measuring at 256 nm). (**B**) Box plots showing different alpha diversity measurements per condition. (**C**) Volcano plot of differential abundance analysis using Maaslin2, comparing MAN against NSC at 24h. Blue colour indicates higher abundance in NSC condition at p < 0.05.


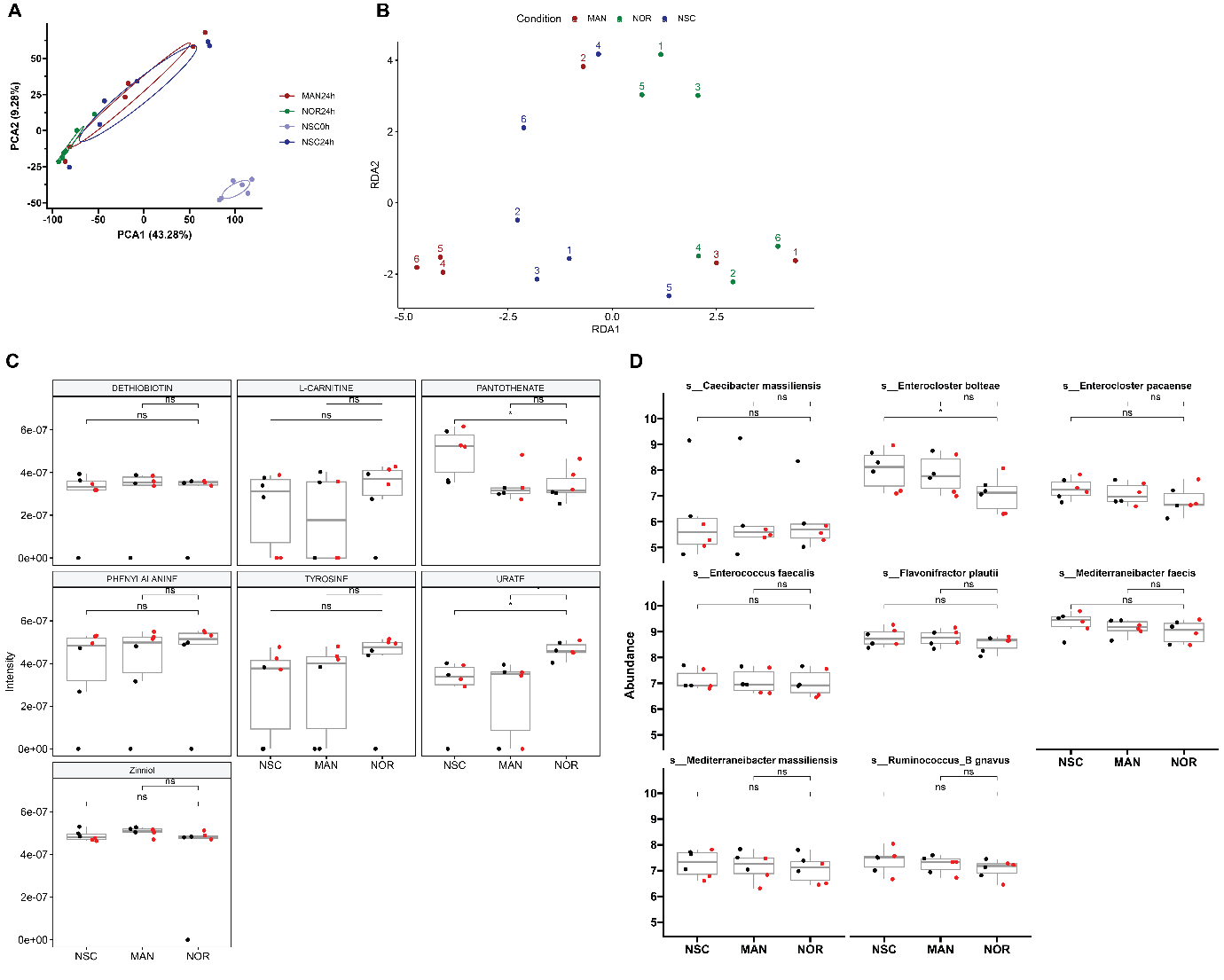


**Supplementary Figure 3**. (**A**) PCA showing clustering of metabolites form the apolar fraction. (**B**) RDA constrained to treatment, similar to **Figure 4C**, showing donor IDs and grouping of two MAN treated samples with converter activity together with NOR treated samples. (**C**) Abundances of metabolites identified to associate with either NOR or NSC from RDA analysis. (**D**) Abundances of species with at least 4 present genes from the urate metabolism gene cluster.
